# Integrated Top-Down Proteomics for Comprehensive Proteome Analysis at the Proteoform Level

**DOI:** 10.3390/proteomes14040053

**Published:** 2026-10-09

**Authors:** Luke A. Farrell, Breyer Woodland, Aiden Brennan, Jens R. Coorssen, Matthew B. O’Rourke, Matthew P. Padula

**Affiliations:** 1 School of Life Sciences, Faculty of Science, University of Technology Sydney, Ultimo, NSW 2007, Australia; lukefarrell98@outlook.com (L.A.F.); breyer-lynne.woodland@uts.edu.au (B.W.); aiden.brennan@uts.edu.au (A.B.); matthew.orourke1@gmail.com (M.B.O.); 2 Institute for Globally Distributed Open Research and Education (IGDORE), St. Catharines, ON L2N 4X2, Canada; jrcoorssen@gmail.com

**Keywords:** 2D-PAGE, proteomics, proteoforms, post-translational modifications, proteogenomics, integrated top-down proteomics

## Abstract

Background: Proteoforms are the critical functional species that drive biological processes. However, conventional proteogenomic (“shotgun”) analyses discard relevant proteoform-level information through bulk protein digestion. This study presents a proof-of-principle integrative Top-Down Proteomics workflow designed to identify and characterise proteoforms across the breadth of a proteome. Methods: This workflow combined two-dimensional electrophoresis, a custom grid-cutting lattice for reproducible gel sectioning, in-gel digestion, liquid chromatography–tandem mass spectrometry, and open-search algorithms. Gel-grid locations, isoelectric points, and molecular weights were integrated with mass-spectrometry results to further characterise identified proteoforms. Results: The approach enabled identification of a broad range of proteoforms and their inherent post-translational modifications, including phosphorylation, acetylation, methylation, and glycosylation. Notably, substantial numbers of proteoforms were identified in visually “blank regions” of replicate two-dimensional gels. This eliminated bias associated with assessing only visually detectable protein spots and substantially expanded proteome-level proteoform detection and characterisation. Conclusions: This integrative workflow provides a reproducible and comprehensive strategy for addressing the often-overlooked complexity of proteomes at the proteoform level. By extending analysis beyond visually detectable gel spots, it enables deeper characterisation of proteoform diversity and associated post-translational modifications.

## 1. Introduction

Proteoforms, the myriad protein species that arise from any given transcribed genome region, include post-translationally modified variants. This speciation from a limited genome accounts for the diversity and regulation of ‘protein’ functions and localizations, and thus drives (molecular) physiology [1,2,3]. Despite widespread recognition of the critical roles of proteoforms, the last ~25 years of proteomics have largely ignored them, using proteogenomic or bottom-up (BUP)/shotgun approaches to analyse abundance changes in the proteome. Rather than analysing intact proteoforms, BUP leverages the resolution of smaller peptides by reversed-phase chromatography and the sensitivity gains in measuring a smaller number of charge states by mass spectrometry (MS) [3]. Consequently, proteogenomics simplifies proteome analysis to one ORF product per gene or a set of ORF products (e.g., ‘protein groups’) when some modified peptides are also identified [4]. This is a significant limitation considering the native proteome complexity (e.g., >680 known post-translational modifications (PTMs)) [5]. However, most PTMs remain largely undetected and/or disregarded in proteogenomic workflows due to low abundance, poor ionisation efficiency or exclusion from the search space criteria [6,7,8,9]. Furthermore, despite often quite limited sequence coverage data from the detected peptides, the routine assumption is that the full-length canonical protein has been identified, ignoring possible size variants or even the presence of a lone peptide [3]. The tendency to promote throughput by using only single analytical ‘runs’ also increases intrinsic error [10]. Thus, without first resolving intact proteoforms, proteogenomics can only infer the presence of potentially intact canonical proteins. Simply put, proteogenomics is incapable of deep, comprehensive proteome analysis at the level of proteoforms. Efforts must shift to methods capable of analysing and characterising proteoforms across the breadth of the proteome, and to further refining and optimising these approaches [1,3].

Top-Down Proteomics (TDP) provides proteoform-level information by resolving intact species prior to identification by MS. TDP methods are either MS intensive (MSi-TDP), typically following a single dimension of reversed-phase chromatography, or utilise a hybrid approach in which proteoforms undergo high-resolution separation using multi-dimensional liquid chromatography (LC) or one- (1DE; typically SDS-PAGE) or two-dimensional gel electrophoresis (2DE), to separate proteoforms prior to peptide- or proteoform-centric MS [1,3,10,11,12]. Once resolved (i.e., the ‘top-down’ step in analysis), proteoforms can be digested to peptides and subjected to LC and tandem MS (LC-MS/MS), an approach known as Integrative Top-Down Proteomics (iTDP) [1,3,13]. While MSi-TDP presents an idealised situation in which intact proteoforms are directly ionised and analysed by LC-MS/MS, this method suffers from significant challenges including substantial limitations to routinely analysing proteoforms > 25 kDa and spectral complexity (including chimeric spectra) that is difficult to deconvolute when assessing total proteome extracts, even with current software tools [3,14]. In contrast, iTDP most often uses 2DE for high-resolution proteoform separation by isoelectric point (pI) and relative molecular weight (MW), thereby resolving proteoforms to single coordinate points or spots in the gel. iTDP is thus able to overcome some limitations of proteogenomics and MSi-TDP as proteoform-level information is established based on in-gel coordinates (i.e., intersection of pI and MW); excision, digestion, and LC-MS/MS then enable identification of both sequence and PTMs, with pI and MW information further confirming a specific proteoform.

Employing the above level of proteoform separation significantly reduces sample complexity prior to LC-MS/MS, resulting in significantly higher proteoform sequence coverage as more copies of the individual peptides are present for analysis [10,11,15,16,17,18,19,20]. However, 2DE is often labelled as low-throughput, and resolved native proteomes yield hundreds or more discrete visual spots that must be individually excised, digested, and analysed by LC-MS/MS. Conventionally, only a handful of spots of interest are analysed based on differential abundance/staining intensity, as excising every spot (i.e., including those not changing in abundance) is time- and instrumentation-intensive. Nonetheless, for such a complete gel analysis, generally only visible (i.e., stained) spots are excised, ignoring those apparently ‘empty’ regions of the gel that may contain resolved proteoforms of low abundance (i.e., below the detection limit of the stain). A deep imaging approach enabled somewhat better detection of lower-abundance species by excising high-abundance spots and re-imaging the gel, thus improving the dynamic range of detection [19,21,22]. However, this approach still has limitations in being a manual process that nevertheless relies on visualisation of stained proteoforms.

Currently, iTDP is the only method capable of providing the unbiased analytical depth needed to assess whole proteomes at the critical proteoform-level [1,3,10,13,23,24,25]. The technique has undergone extensive method development, optimisation, and refinement, from sample preparation through the 2DE protocol and subsequent analysis, that overcomes many of the (implied) early limitations of the technique [11,20,21,22,25,26,27,28,29,30,31,32,33,34,35,36,37]. The current robustness of the method coupled with the routine approach of resolving replicate gels per sample ensures reproducibility and data integrity. Nonetheless, a lack of broader automation relative to proteogenomic experiments continues to deter more widespread use of iTDP. Here, building on established methods [38,39], we present a more refined approach to whole proteome analysis of proteoforms via a new grid-cutting device to uniformly divide a whole 2DE gel into small sections containing separated proteoforms that are then analysed, identified and characterised by peptide-centric LC-MS/MS. Coupled with an open PTM search, this enables a thorough approach that addresses critical proteoform-level information. Although manual sectioning of 2D gels has previously been used to enable deeper proteome analysis [38,39], this study introduces a simple proof-of-principle approach using a 3D-printed grid-slicing tool that enables direct deposition of gel sections into a 96-well plate for further processing prior to LC-MS/MS. Overall, this iTDP approach effectively characterises proteoforms through their gel coordinates (i.e., pI and MW), sequence, and identified PTMs.

## 2. Materials and Methods

All reagents were of analytical and electrophoresis grade or higher. The following chemicals and reagents were purchased from Sigma Aldrich (St. Louis, MO, USA): Urea, Thiourea, C7BzO (3-(4-Heptyl)phenyl-3-hydroxypropyl)dimethylammoniopropanesulfonate), Tris-HCl, tris(2-carboxyethyl)phosphine (TCEP), Roche cOmplete™ Mini EDTA-free Protease Inhibitor (PI) Cocktail tablets, sodium n-dodecyl sulphate (SDS), bromophenol blue, glycerol, phosphoric acid, methanol, ammonium sulphate, citric acid, glacial acetic acid, ammonium bicarbonate, and Bovine Serum Albumin (BSA) for gel assays (product no. A7517, lot no. SLCM2607). BeadBug^TM^ homogenization tubes (2 mL capacity) and 3 mm zirconium beads from Benchmark Scientific (Sayreville, NJ, USA) were purchased from Sigma Aldrich. The following were purchased from Bio-Rad laboratories (Hercules, CA, USA): 2D electrophoresis equipment, ReadyStrip™ IPG Strips (7 cm, pH 3–10 non-linear), 40% acrylamide/bis-acrylamide (37.5:1) solution, Precision Plus Protein™ Unstained Standards (10–250 kDa), 15-well Mini-PROTEAN^®^ TGX™ Acrylamide gels used for total protein assays, Coomassie Brilliant Blue (CBB) G250, acrylamide monomer powder, and low melt agarose. Trypsin Gold sequencing grade was purchased from Promega (Promega, Madison, WI, USA). Skirted low profile 96-well PCR plates and mass spectrometry grade 100% *v*/*v* acetonitrile (ACN) were purchased from ThermoFisher (Waltham, MA, USA). Axygen 2 mL deep squared 96-well square plates and sealing mats were purchased from Pacific Laboratory Products (Blackburn, VIC, Australia). All 3D-printing equipment and consumables (Polyethylene Terephthalate Glycol filled with Carbon Fibre (PETG-CF), and Polylactic acid (PLA)) to print the gel cutting lattice were purchased from BambuLab (Shenzhen, China). STop And Go Extraction (STAGE) tips were made using double-stacked polystyrene–divinylbenzene, reversed-phase sulfonate (SDB-RPS; 3M) discs purchased from Sigma-Aldrich cut with an 18-gauge needle before insertion into a 200 μL pipette tip [40].

### 2.1. Tissue Homogenisation and Protein Extraction

Three livers were dissected from mice that were culled for reasons unrelated to this study by the Ernst Facility, University of Technology Sydney. Immediately following collection, the tissue was washed in ice cold sterile phosphate-buffered saline (PBS) to remove blood, with the first wash containing 1X PI, until the wash solution did not have a pinkish-red tone. The washed tissue was diced and transferred to a homogenisation tube, and 500 µL of homogenisation buffer (7 M urea, 2 M thiourea, 50 mM Tris-HCl (pH 8.8), and 1X PI) was added. The sample was homogenised using a BeadBug™ benchtop homogeniser (Benchmark Scientific, Sayreville, NJ, USA) at 4000 rpm for 30 s for 4 rounds, with cooling on ice between rounds. C7BzO was added to a final concentration of 1% (*w*/*v*) after homogenisation, samples were combined, diluted to a total volume of 20 mL, and probe sonicated using a Qsonica Q125 Sonicator (Qsonica, LLC, Newtown, CT, USA ) with a CL-18 probe at 40% amplitude for 6 rounds of 30 s on/30 s off, and placed on ice during ‘off’ periods. The sample was reduced and alkylated simultaneously with 5 mM TCEP and 10 mM acrylamide monomers at room temperature for 1 h.

Extracted proteins were precipitated with five volumes of ice-cold acetone and incubated overnight at −20 °C, followed by centrifugation at 3214× *g* for 5 min. The acetone was discarded and the protein pellet was air dried in a fume hood prior to resuspension in 20 mL UTC7 (7 M urea, 2 M thiourea, 1% C7BzO, and 1X PI). Insoluble material (e.g., connective tissue) was removed by centrifugation at 3214× *g* for 5 min and the supernatant was transferred to a new tube prior to repeating acetone precipitation, resuspension and centrifugation to ensure optimal extraction. Protein quantification was performed using a gel-based assay with BSA as a standard [41].

### 2.2. 2DE: IEF and SDS-PAGE

IEF and SDS-PAGE were carried out as previously described with minor changes [29]. Three gel replicates were resolved in parallel (Supplementary Figure S1). Briefly, 7 cm non-linear pH 3–10 IPG strips were micro-perforated to enable rapid rehydration and then rehydrated with 100 μg of protein for 4 h at RT [26]. Using a Bio-Rad PROTEAN IEF cell, rehydrated strips were overlaid with mineral oil and focused using the following programme: initial desalting phase at 250 V for 15 min, followed by a linear ramp from 250 V to 4000 V over 2 h, and finally held at 4000 V until 37,500 Vh was reached.

Focused IPG strips were then placed in equilibration buffer (6 M urea, 2% *w*/*v* SDS, 0.375 M Tris-HCl (pH 8.8), 20% *v*/*v* glycerol) for 20 min. SDS-PAGE was carried out as previously described using 12%T mini-format gels [32]. Gels were fixed with 1 M citric acid and 5% *v*/*v* acetic acid for 1 h, then washed in Milli-Q water (3 × 20 min washes) before staining in colloidal Coomassie brilliant blue (cCBB; 10% *w*/*v* ammonium sulphate, 2% *v*/*v* phosphoric acid, 0.1% *w*/*v* Coomassie G250, 20% *v*/*v* methanol) overnight [28].

cCBB stain was disposed of, and the gel was briefly washed in 40% *v*/*v* methanol, 10% *v*/*v* acetic acid for 30 s to remove excess stain, then washed with 0.5 M sodium chloride to destain until the background was colourless [42]. Following destaining, gels were imaged by near-infrared fluorescence detection (nIRFD) using a GE Healthcare Typhoon FLA 9000 Biomolecular Imager with 685 nm excitation laser, 713–726 nm emission filter (BPFR700, GE Healthcare, Chicago, IL, USA), 50 μm pixel size, and PMT gain set to 600 V [32]. Gels were analysed using Delta2D v4.8.2 (DODECON, Waldenbuch, Germany).

### 2.3. Gel Slicing

Gels were oriented with the basic side (i.e., pH 10) of the gel aligned with column 1 on a square, deep-well 96-well plate. To ensure reproducible alignment and thus that proteoforms were being cut into the same grid/well coordinate, the grids of the deep-well plate were aligned with well-resolved high-abundance spots to act as reference points. This resulted in 63 of the 96 wells being covered by the gel. A cutting lattice was then pressed over the top of the gel, slicing the gel into 63 grid sections/samples. The lattice was left in situ while centrifuging the assembly at 1000× *g* for ~30 s, transferring all the gel sections into the bottom of the underlying well, thus allowing them to subsequently be fully submerged in digestion buffers. For a detailed visual overview of the gel slicing apparatus and method, see Supplementary Figure S2.

### 2.4. In-Gel Digestion and Sample Clean-Up

Sliced gel grids were immediately washed twice in destain solution (50% (*v*/*v*) ACN/50 mM ammonium bicarbonate [pH 9]) for 10 min. Gel grids were then dehydrated with 800 μL of 100% *v*/*v* ACN each and agitated on a rocker for 30 min. After incubation, all liquid was removed and discarded. Following dehydration, each gel piece was rehydrated with 50 μL of trypsin in-gel digest solution (3 ng/µL trypsin in 100 mM ammonium bicarbonate), followed by ‘short spin’ centrifugation (i.e., a brief pulse to quickly spin gel pieces to the bottom of the well) to ensure full contact with solution. The plate was covered with a sealing mat and incubated at 4 °C for 30 min, followed by addition of 50 μL of 100 mM ammonium bicarbonate to each well. The plate was briefly centrifuged again, then incubated at 37 °C for 16 h.

Following digestion, the plate containing the gel pieces was bath sonicated using a Thermoline Scientific Power Sonic240 (Thermoline Scientific, Wetherill Park, NSW, Australia.) on the high frequency setting for 10 min prior to adding 250 μL of SPE load solvent (90% *v*/*v* ACN, 1% *v*/*v* TFA) to each well. The plate was agitated on a rocker until all gel pieces had fully dehydrated (approximately 30 min), allowing the peptides to diffuse out of the gel and into solution.

The peptide-containing solutions were then transferred into 63 self-made STAGE tips using a multi-channel pipette [40]. STAGE tips were mounted into a 3D-printed apparatus designed by Harney et al. and printed from PETG-CF using a Bambu Labs X1C (Shenzhen, China) [43]. STAGE tipping was then performed as previously described [44]. The 96-well capture plate was then placed in a Savant^TM^ DNA 120 SpeedVac Concentrator (Thermo Fisher Scientific, Waltham, MA, USA), and the samples were evaporated to dryness on the medium drying rate setting. The plates were then covered and placed at −20 °C until analysis. In preparation for MS analysis, samples were resuspended with 5 μL of MS Loading solvent (2% *v*/*v* ACN and 0.1% *v*/*v* TFA) and manually transferred to glass vial autosampler inserts.

### 2.5. iTDP Mass Spectrometric Analysis

Using an Acquity M-class nanoLC system (Waters, Milford, MA, USA), 5 μL of sample was loaded at 15 μL/min for 2 min onto a nanoEase Symmetry C18 trapping column (180 μm × 20 mm) before being washed with 97% MS buffer A (0.2% *v*/*v* formic acid in water) and 3% MS buffer B (100% *v*/*v* ACN) onto an Aurora Rapid XT C18 column (80 mm × 150 μm ID, 1.7 μm C18 particle size; Ion Opticks, Fitzroy, Australia) heated to 45 °C. Peptides were eluted from the column at a flow rate of 1 μL/min and into the source of a Q Exactive Plus mass spectrometer (Thermo Scientific, Waltham, MA, USA) using the following MS buffer B gradient: 10–40% over 15 min, 40–80% over 30 s, 80% for 30 s, 80–5% over 30 s, and 5% for 4.5 min. The eluting peptides were ionised at 2100 V. A Data Dependent MS/MS (dd-MS2) experiment was performed, with a survey scan of 350–1500 *m*/*z* at 70,000 resolution for peptides of charge state 2+ or higher with an AGC target of 3 × 10^6^ and maximum injection time of 50 ms. The top 12 peptides were selected and fragmented in the HCD cell using an isolation window of 1.4 *m*/*z*, an AGC target of 1 × 10^5^ and maximum injection time of 100 ms. Fragments were scanned in the Orbitrap analyser at 17,500 resolution and the product ion fragment masses measured over a mass range of 120–2000 *m*/*z*. The mass of the precursor peptide was then excluded for 5 s. Interspersed between analyses of the individual gel grid digests, 5 μL of ‘clean solution’ (1:1:1:1 water/ACN/methanol/isopropanol) was loaded and a blank injection was performed to limit peptide carry-over between sample injections.

### 2.6. Proteogenomic Analysis

To compare this iTDP gel-based method to standard proteogenomic-based analyses, 100 μg of the same total liver protein extract was diluted 10-fold with 100 mM Tris-HCl to reduce the concentration of urea to <1 M and proteoforms were digested with 1 μg of sequencing grade trypsin at 37 °C for 16 h. Peptides were recovered using SDB-RPS-based STAGE tips. Eluted peptides were evaporated to dryness and reconstituted in 100 µL of MS loading solvent (2% *v*/*v* ACN, 0.2% *v*/*v* trifluoroacetic acid) for a final concentration of 100 ng/µL.

### 2.7. Proteogenomic Mass Spectrometric Analysis

Using an Acquity M-class nanoLC system (Waters, MA, USA), 500 ng in 5 µL of the sample was loaded at 15 μL/min for 2 min onto a nanoEase Symmetry C18 trapping column (180 μm × 20 mm) before being washed onto an Aurora Elite TS C18 column (150 mm × 75 μm ID, 1.6 μm C18 particle size; Ion Opticks, Fitzroy, Australia) heated to 45 °C. Peptides were eluted from the column and into the source of a Q Exactive Plus mass spectrometer (Thermo Scientific) using the following gradient: 5–30% MS buffer B (98% *v*/*v* ACN +0.2% *v*/*v* formic acid) over 120 min, 30–80% MS buffer B over 2 min, 80% MS buffer B for 2 min, and 80–5% for 2 min. The eluting peptides were ionised at 2400 V. A Data Dependent MS/MS (dd-MS2) experiment was performed as described above in Section 2.5.

### 2.8. Data Analysis

The MS/MS raw files were searched using Peaks Studio 13 (Bioinformatic Solutions Inc., Waterloo, ON, Canada) against the *Mus musculus* (Mouse) Unreviewed (TrEMBL) isoform UniProt database (UP000000589, downloaded 10 September 2025) and a database of common contaminants with the following parameters: Precursor mass error tolerance: 10.00 ppm. Fragment mass error tolerance: 0.02 Da. Enzyme: Trypsin. Maximum missed cleavages: 4. Digest-mode: Semi-specific. Peptide length range: 6–45. Fixed modifications: none. Variable modifications: All (i.e., Peaks PTM Open Search). Maximum variable PTMs per peptide: 5. Peptide spectrum match (PSM) false discovery rate (FDR): 1.0%. Protein Group FDR: 1.0%. Unique peptides: ≥1. To confidently determine modification sites, the modified peptide must have an A-score, the localization score assigned to modifications on the peptide, greater than or equal to 15 (*p* value < 0.0316) and an ion intensity ≥ 2 percent of the highest peak in the spectrum. Protein groups reported in database search results consist of “top” proteins, defined as the highest-scoring ORF product within a group of ORF products that share common peptides, ORF products defined as “true” where the search algorithm deems a confident identification due to high scoring with at least one unique peptide, or “false” as ‘redundant’ ORF products supported only by shared peptides.

Exports from Peaks Studio search results were processed in R Studio (version 2025.09.1) using tidyverse packages (v2.0.0) [45]. All figures were created in R Studio using ggplot2 (v4.0.3) and patchwork(v1.3.2) [45]. Welch’s unpaired two-sample t-test with Benjamini–Hochberg (BH) correction was performed in R to determine if there was a significant difference in the number of proteins and peptides identified and average sequence coverage (%) between the iTDP and standard proteogenomic analyses, where an adjusted *p*-value < 0.05 was considered statistically significant. MW and pI were calculated in R studio using the sum of masses of amino acid residues in a sequence and the European Molecular Biology Open Software Suite (EMBOSS) pKa scale from the Peptides package (v2.4.6), respectively [46]. For iTDP grid analyses, potential artifactual PTMs, including oxidation, dioxidation, sulphone, deamidation, dehydration, propionamide, carbamidomethylation-related artefacts, pyro-glutamate, cyano, and carbamylation, were filtered prior to sequence visualisation.

## 3. Results

**Figure 1:**
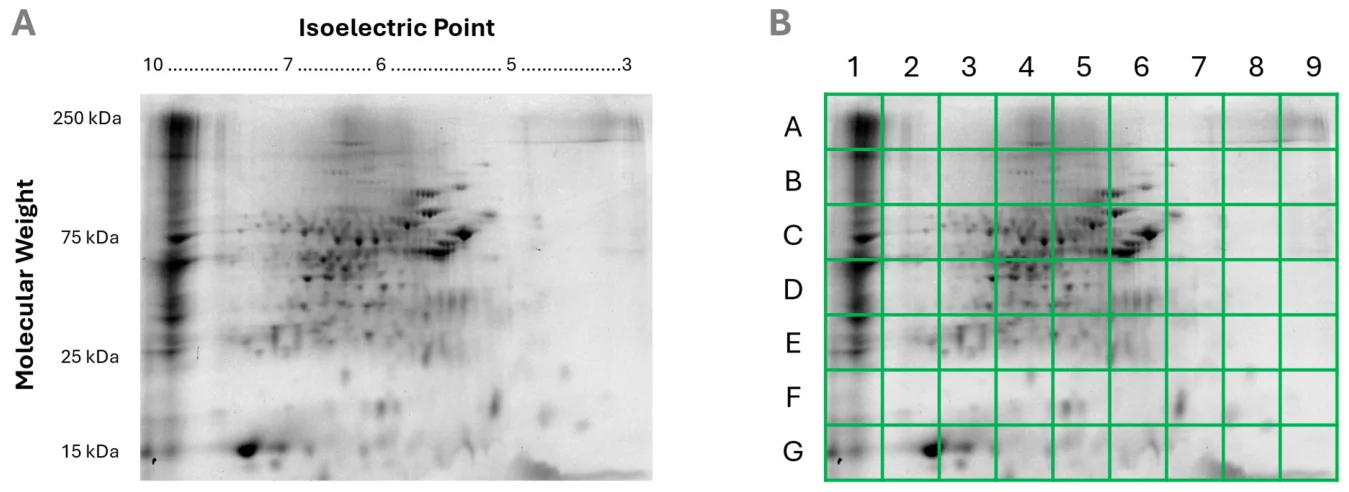
Representative 2DE gel images of mouse liver. (**A**) A total of 100 μg mouse liver proteome extract resolved by IEF on a 7 cm 3–10 non-linear IPG strip and then by SDS-PAGE using a 12% resolving gel, subsequently stained with cCBB (n = 3). (**B**) Representative image of the gel grid and fractions created by grid slicing prior to in-gel digests and LC-MS/MS analysis (n = 3).

Mouse liver proteome extract was resolved by 2DE and stained for total proteoform detection using colloidal Coomassie Brilliant Blue (cCBB) (Figure 1A). A total of 747 ± 18 spots were detected across three replicate gels, establishing the resolution and reproducibility of the method (Supplementary Figure S1). The 3D-printed grid-cutting lattice was used to uniformly cut each gel into 63 sections, allowing every proteoform in the gel to be extracted for LC-MS/MS (Figure 1B); see Supplementary Data File S1 for the approximate MW and pI range of each grid.

### 3.1. ORF Product Detection and Reproducibility

**Figure 2:**
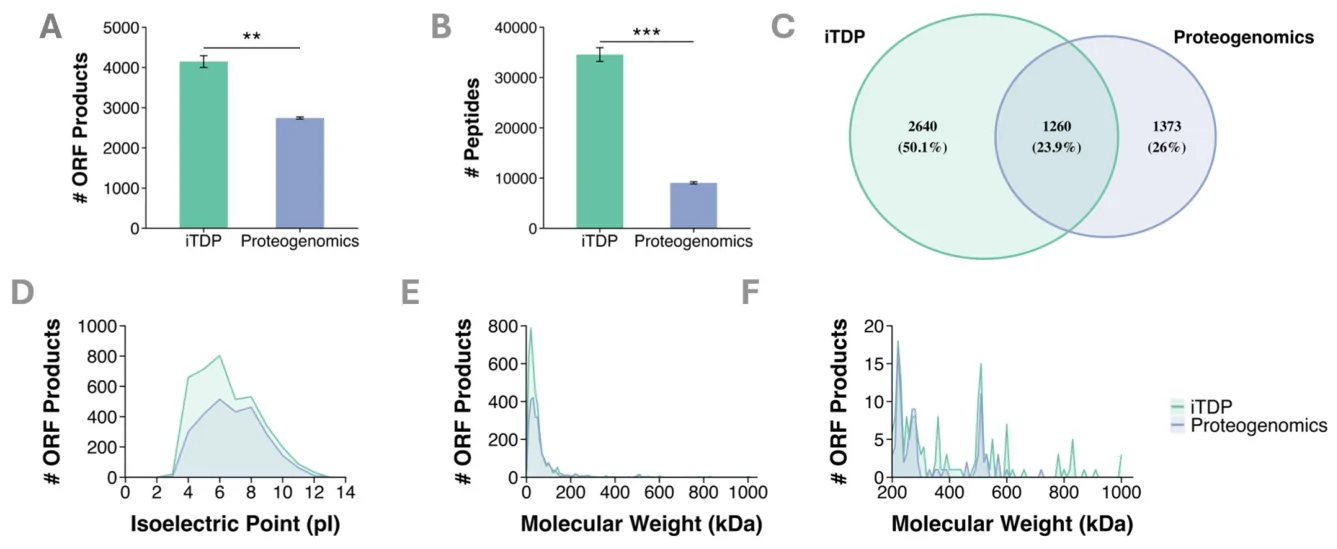
Number of ORF products and peptides identified by iTDP vs. proteogenomics, and physio-chemical properties of unique ORF products identified by each method. (**A**) All ORF products identified with at least one unique peptide and at least one peptide per protein ID (i.e., accession number/ORF product). (**B**) Number of peptides identified by each method. Statistically significant differences are indicated by ‘*’ where two and three symbols indicate BH-corrected *p* < 0.01 and *p* < 0.001, respectively, (Welch’s unpaired two-sample *t*-test, n = 3). (**C**) Venn diagram of all ORF products identified in at least two replicates of each method. (**D**) Isoelectric point of all ORF products identified by each method. (**E**,**F**) Molecular weight distribution of all ORF products identified by each method.

Data from the grid-slicing iTDP method were compared to that obtained using a routine proteogenomic workflow to assess the number of ORF products and peptides detected, depth of sequence coverage, and the critical capacity to characterise proteoforms (Figure 2 and Supplementary Data File S2). Using a one peptide minimum cut-off per protein ID, iTDP identified 4147 ± 147 ORF products vs. 2743 ± 28 using proteogenomics (Figure 2A). Furthermore, when comparing IDs at the peptide level, iTDP enabled a four-fold greater detection, with 34,574 ± 1368 vs. 9055 ± 220 peptides by proteogenomics (Figure 2B). The stark contrast between the two methods, even when increasing the exclusion criteria to two or more peptides per protein ID, indicates that iTDP (3411 ± 44 ORF products) with simple grid-cutting significantly improves the depth of proteome analyses compared to a standard proteogenomic (1873 ± 36 ORF products) approach.

To ascertain potential underlying biases between the two methods, the IDs from the iTDP and proteogenomics approaches were compared (Figure 2). The physicochemical properties of the identified canonical ORF products, specifically pI and MW, were analysed to determine if either method favoured specific protein classes (Figure 2D–F). Overall, minimal differences were observed in these canonical properties.

**Figure 3:**
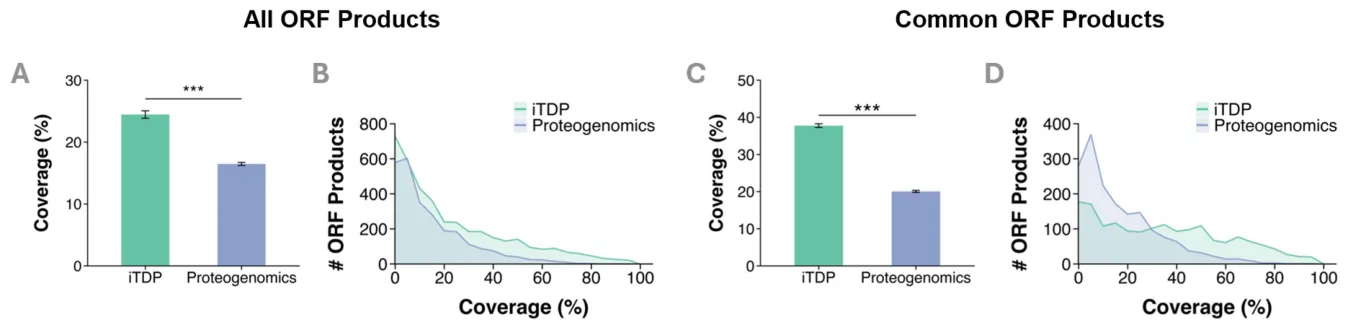
(**A**) Average sequence coverage of all ORF products identified by each method. (**B**) Coverage distribution of all ORF products identified by each method. (**C**) Average sequence coverage of identified ORF products common to both methods. (**D**) Coverage distribution of the common ORF products. Statistically significant differences are indicated by ‘*’ where three symbols indicate BH-corrected *p* < 0.001 (panel (**A**,**C**), Welch’s unpaired two-sample *t*-test, n = 3).

To assess the depth of proteome analyses, we compared the average sequence coverage of total and common ORF products identified by each method (Figure 3). The average sequence coverage of all ORF products identified was 24.4 ± 0.6% and 16.5 ± 0.2% by iTDP and proteogenomic methods, respectively (Figure 3A). When comparing the sequence coverage of ORF products common to both datasets, the averages were 37.8 ± 0.5% and 20.1 ± 0.3% for iTDP and proteogenomics, respectively (Figure 3C). Thus, by both metrics, iTDP clearly provided significantly increased ORF sequence coverage, almost doubling that of proteogenomics for common IDs. Additionally, a significantly greater proportion of ORF products identified by iTDP had >60% sequence coverage (Figure 3B,D). Higher coverage also enabled higher scoring between protein variants that share peptides, thereby increasing confidence in discrimination between related species. For example, in sample iTDP_03, grid location A8, Histone H3.3 and its fragment (accession no. P84244 and E0CZ27) were identified as the “true” ORF products in the grid location, with six additional related entries classified as “false” due to shared peptides (Supplementary Table S1). With 68.4% sequence coverage, P84244 could be confidently distinguished from potential variants and closely related proteins due to its substantially higher protein score with the one drawback being the inability to determine if it or its slightly (17 residue) shorter fragment was present in this location due to shared peptides. In contrast, in the proteogenomic analysis (Proteogenomics_02), the same ORF product was detected; however, with only 31.6% coverage (Histone H3.3: P84244), two other potential ORF products (Histone H3.3 protein fragment: E0CZ27 36.1%, H3.3 histone A: P02301 31.6%) remain the plausible “true” proteins because they share similar scores and cannot be definitively differentiated (Supplementary Table S1).

### 3.2. Assessment of Proteoform-Level Identification

Although shotgun assessments can detect modified peptides that are then classified into ‘protein groups’ together with the identified canonical sequence, all information as to intact proteoforms is lost. In contrast, using high-resolution front-end separation, iTDP can distinguish between proteoforms arising from the same ORF product.

With iTDP, the presence of a proteoform is indicated by identification of an ORF product at a specific gel grid coordinate. The same ORF product being identified at different coordinates indicates that multiple proteoforms are present, without the need to definitively identify the nature of each unless necessary. If each ORF product found in a different gel grid is a unique proteoform, and there are no overlapping spots between two gel grids (i.e., a spot ‘cut in half’), the iTDP method detected 2574 ± 49 ‘protein groups’ (see Section 2.8), 4147 ± 147 total ORF products (i.e., all ‘top’ ranked proteins) which consisted of a total of 29,075 ± 654 proteoforms, corresponding to a total of 2621 ± 51 genes (Supplementary Data File S2). The proteogenomic method only detected 1593 ± 15 ‘protein groups’, 2743 ± 28 total ORF products, which linked back to a total of 1656 ± 11 genes; standard proteogenomic limitations made proteoform ID impossible.

**Figure 4:**
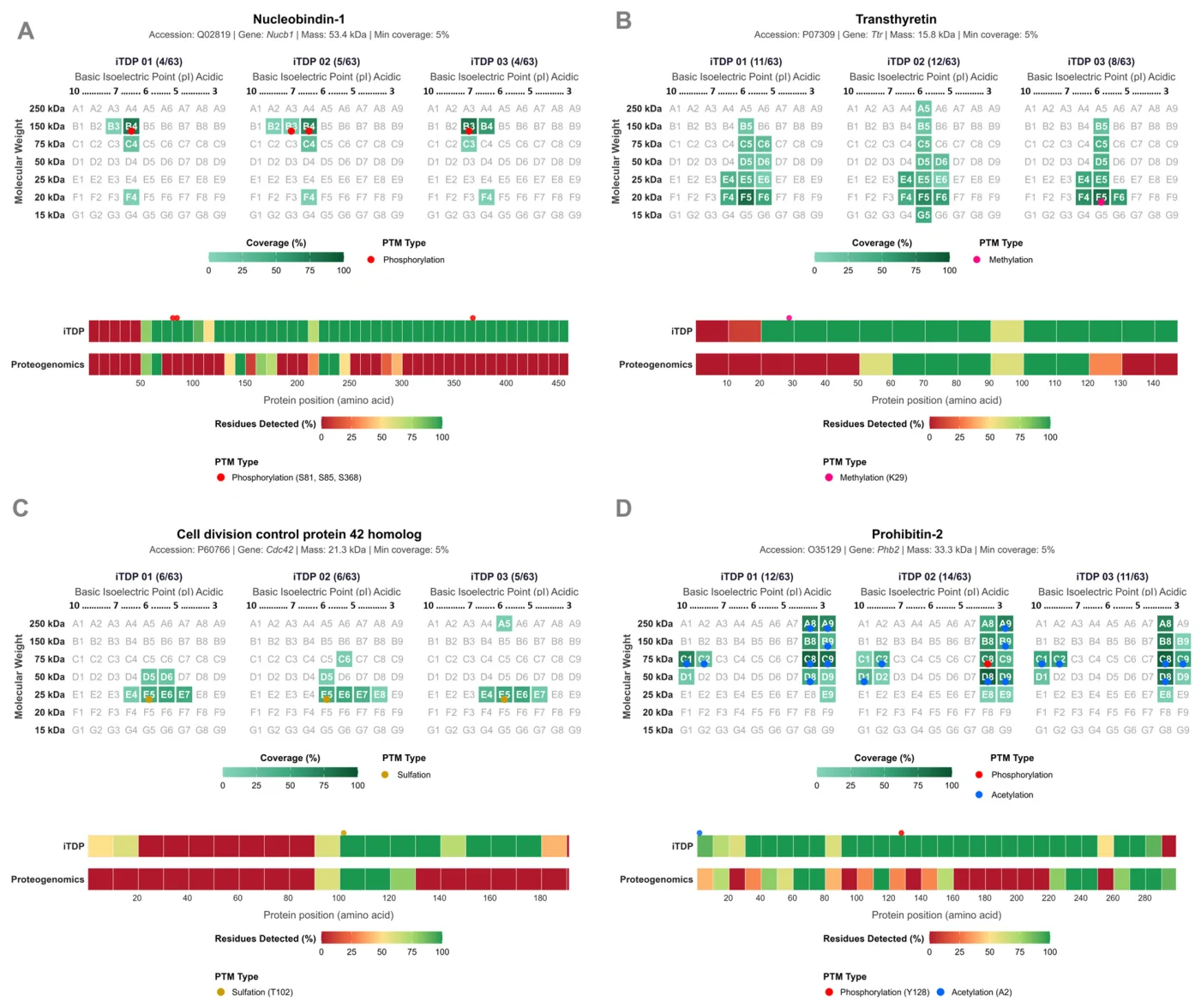
Combined iTDP grid and linear sequence coverage/PTM visualisation for (**A**) nucleobindin-1, (**B**) transthyretin, (**C**) cell division control protein 42 homologue, and (**D**) prohibitin-2. (**A**–**D**) iTDP 2D-PAGE grid occupancy across iTDP 01–03 together with linear residue coverage and confidently localised PTM sites, with bin colour representing residues detected (%) and coloured dots denoting confidently localised PTM sites. Minimum coverage threshold was 5% for all proteins.

To further assess the capacity of iTDP to identify proteoforms, four ORF products identified by both iTDP and proteogenomics were selected for more detailed analysis (Figure 4). For Nucleobindin-1, despite the ORF product being confidently identified by proteogenomics with coverage of 15.8 ± 2.5%, the peptide defining the proteoform with phosphorylated sites (S81/S85, S368) was absent, even in its unmodified form. The modified peptide that defines the respective proteoforms was detected by iTDP, with a global coverage of 79.2 ± 7.6%, and the modified proteoforms found in grids B3 and 4 (Figure 4A). This was also the case for the methylation of Transthyretin (K29) with proteogenomics yielding 44.2 ± 0% coverage but being unable to detect the modified proteoform-defining peptide, while iTDP detected the peptide in 1/3 replicates, with sequence coverage of 83.9 ± 0.4% (Figure 4B). In the case of cell division control protein 2 homologue, proteogenomics yielded 17.4 ± 0.6% coverage but did not detect proteoform-defining modifications, whereas iTDP achieved 50.2 ± 1.0% coverage and detected sulfation at T102 (Figure 4C). For Prohibitin, proteogenomics yielded a coverage of 32.6 ± 6.2% yet was unable to detect modified peptides. In contrast, iTDP achieved 91.0 ± 0% coverage and detected the acetylation of Alanine at position 2, the mature proteoform N-termini after methionine removal, in all replicates and the phosphorylation of Y128 in 1/3 replicates (Figure 4D). Overall, the data consistently indicate the inherent capacity for iTDP to identify proteoforms.

**Figure 5:**
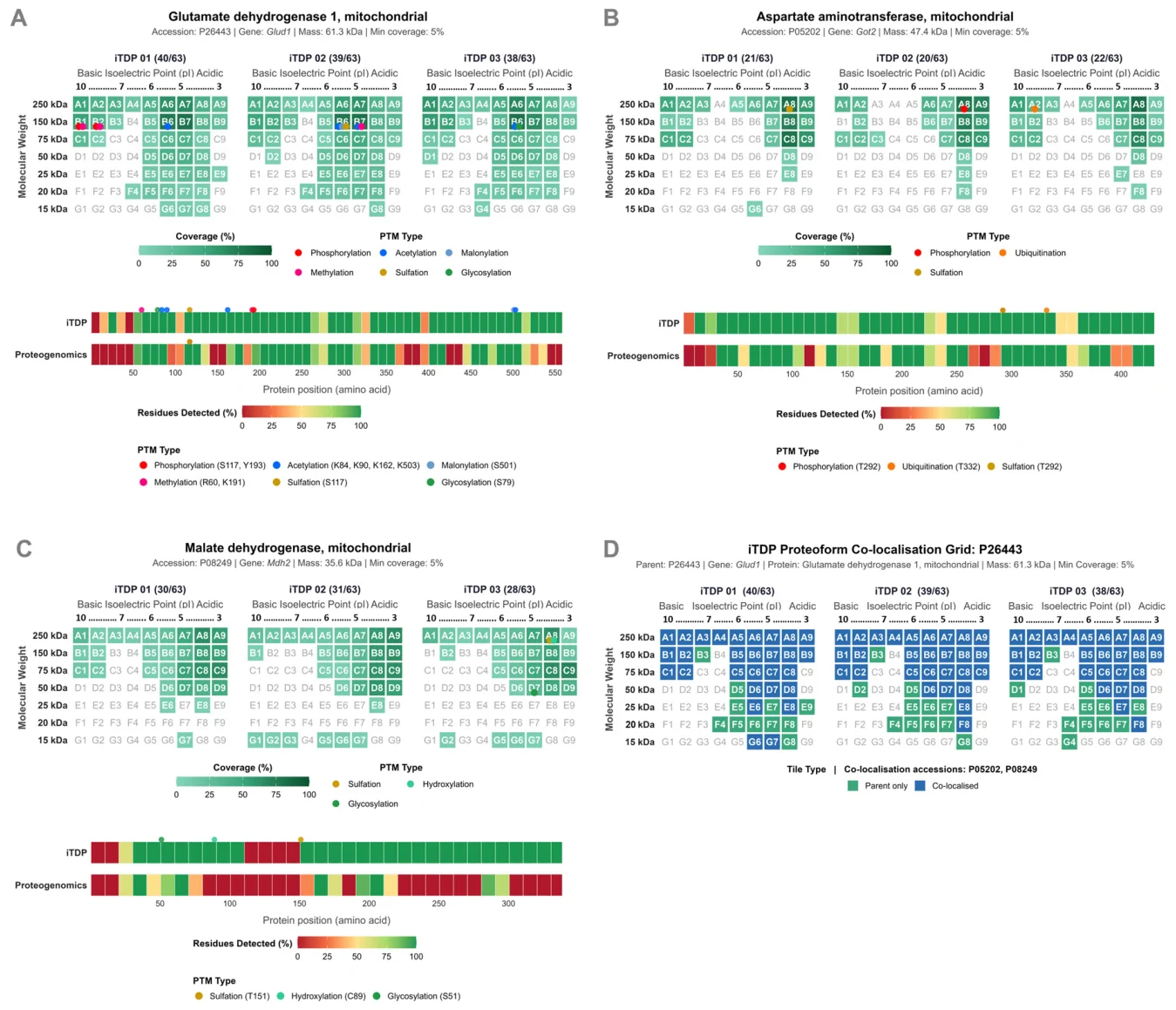
Combined iTDP grid and linear sequence coverage/PTM for (**A**) glutamate dehydrogenase 1, mitochondrial, (**B**) aspartate aminotransferase, mitochondrial, and (**C**) malate dehydrogenase, mitochondrial, and ORF product co-localisation visualisation (**D**). (**A**–**C**) iTDP 2D-PAGE grid occupancy across iTDP 01–03 together with linear residue coverage and confidently localised PTM sites, with bin colour representing residues detected (%) and coloured dots denoting confidently localised PTM sites. D: Co-localisation of P26443 with P05202 and P08249 across the same gel grids. Minimum coverage thresholds were 5% for all three proteins.

Thus, comparable observations were made regarding Glutamate Dehydrogenase-1 (GDH-1; Figure 5A), Aspartate aminotransferase (Figure 5B), and Malate Dehydrogenase (Figure 5C). GDH-1 had 84.53 ± 1.67% coverage by iTDP, compared to 60.39 ± 2.15% by proteogenomics. Several proteoform-defining modifications (acetylation K84, K90, K162, K503, glycosylation S79, malonylation S501, Methylatyion R60, K191 and phosphorylation S117, Y193) were exclusively detected by iTDP, yet proteogenomics detected only the unmodified peptides, with the one exception being the sulfation/phosphorylation of S117. As phosphorylation and sulfation are isobaric modifications, this finding requires further experimentation to determine the true species present [47]. Despite high sequence coverage of the ORF product by both iTDP and shotgun methods, the latter was not capable of identifying most PTMs found by iTDP and, importantly, PTMs defining a particular proteoform. Again, iTDP enabled definitive and comprehensive proteoform characterisation.

Furthermore, of the detected modifications presented here, only those present on Prohibitin-2 (acetylation of A2 and phosphorylation of Y128), and the acetylations on GDH-1 (at K84, K90, K162 and K503) were reported as known modifications in UniProt, with the remaining PTMs representing potential novel modifications requiring further validation.

### 3.3. Detection of Proteome-Wide Post-Translational Cleavage and Proteoform–Proteoform Interactions

Numerous proteoforms resolve in MW regions significantly higher than the theoretical MW of their respective ORF product (Figure 5). For example, GDH-1 has a theoretical MW of 61.3 kDa, yet proteoforms were detected in areas of the gel corresponding to MW > 150 kDa. Interestingly, GDH-1 was detected across the entire resolved MW range in a reproducible manner between gel replicates (Figure 5A). GDH-1 is reported to exist as a homohexamer, putting its native multimeric size at ~370 kDa [48]. While these could represent heavily modified species, the substantial difference relative to the theoretical MW more strongly suggests complexation. Thus, we propose that the detection of smaller ORF product sequences in high MW regions may be due to protein–protein interactions that are not susceptible to denaturation or disruption with the chaotropes, surfactants and reducing agents used during the extraction process and are unable to be detected by proteogenomics. To further analyse the potential of GDH-1 interacting with other proteoforms, we assessed the presence of potential binding partners previously reported in the literature, including Malate dehydrogenase and Aspartate aminotransferase [49,50]. Indeed, proteoforms of Malate dehydrogenase and Aspartate aminotransferase resolved to most gel locations where GDH-1 proteoforms also resolved, indicating potential interactions that were not disrupted by the current sample handling/preparation protocol (Figure 5).

**Figure 6:**
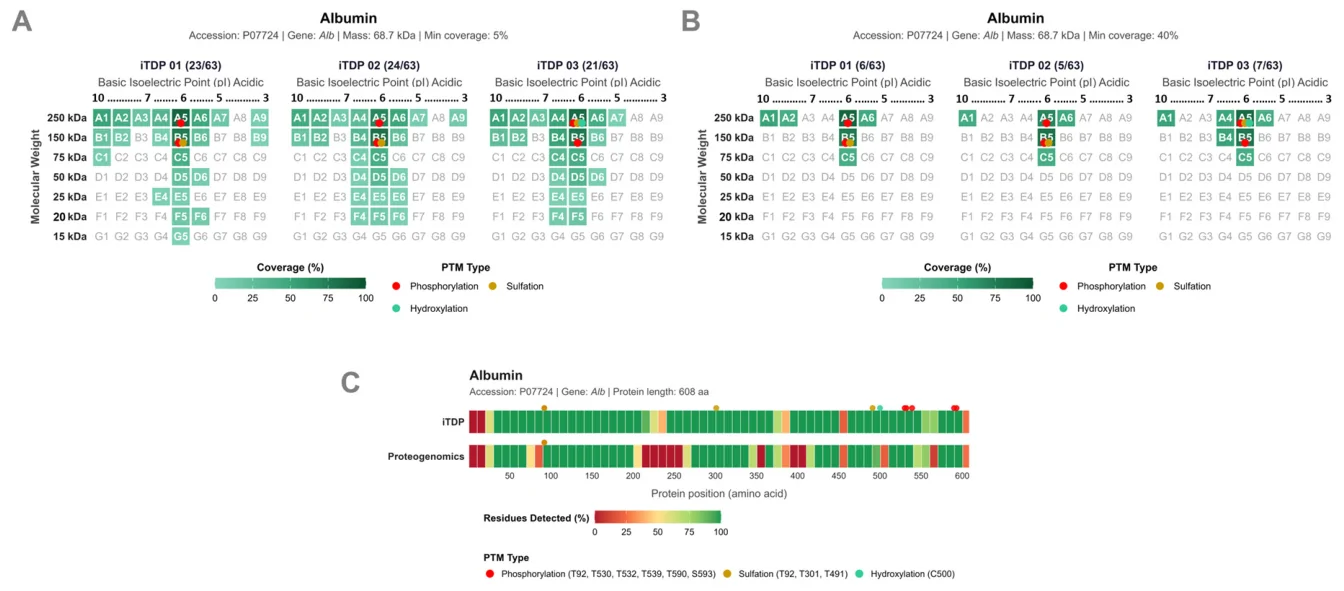
iTDP grid and linear sequence coverage/PTM visualisation for albumin (P07724). (**A**,**B**) iTDP 2D-PAGE grid occupancy across iTDP 01–03 using minimum coverage thresholds of 5% and 40%, respectively. (**C**) linear sequence coverage for iTDP and proteogenomic datasets, with bin colour representing residues detected (%) and coloured dots denoting confidently localised PTM sites.

Overall, this iTDP grid sectioning approach demonstrated the reproducible separation and resolution of proteoforms from a complex native proteome. To further confirm reproducibility, we compared the resolution of albumin proteoforms in the mouse liver extract to that of 2DE-resolved proteoforms previously identified by direct spot cutting [44]. Here, iTDP identified a similar pattern of albumin proteoform resolution from the total liver protein extract as albumin proteoforms found in commercially isolated BSA samples. In both data sets, albumin was detected in a range of locations in the gel due to numerous PTMs, including phosphorylation (Figure 6). When examining the distribution of albumin proteoforms (using a minimum sequence coverage of 5%), a diversity of proteoforms was detected (23 grid locations/proteoforms ± 1.5), including in low MW regions that may indicate proteolytic products (Figure 6). When increasing the minimum coverage threshold to 40%, higher-abundance albumin proteoforms were mainly identified (6 grid locations/proteoforms ± 1). Consistent with previous analyses, a large portion of these high-abundance proteoforms were present at a significantly higher MW than that of albumin monomers [44].

Overall, we identified a significantly higher portion of ORF products (i.e., canonical proteins) through iTDP, with significantly more peptides and sequence coverage, paired with a significant increase in modified peptides detected. Another key finding was that, despite the lack of observable protein staining in several gel locations, a multitude of ORF products were detected by LC-MS/MS (i.e., sections A3,6,7; B3,7–9; C7–9; D2,7–9; E7–9; F2,8,9; G8,9. Refer to Figure 1B). For example, in the seemingly ‘empty’ location of B9 for all gel replicates, an average of 1137 ± 515 peptides, 398 ± 105 top proteins, and 285 ± 72 protein groups from 295 ± 74 genes were detected (Supplementary Data File S2). This trend of identifying proteoforms in seemingly ‘empty’ gel locations was also documented in every other location where spots could not be visually detected (see Figure 1B and Supplementary Data File S2).

## 4. Discussion

With the increasing recognition of proteoforms as the functional molecular units, the abundance of which must be measured to understand biological changes, focus must shift to identifying the full range of proteoform diversity and away from non-discriminating high-throughput proteogenomics [1,3]. The data here highlight a method for the comprehensive analysis of proteoform diversity in complex proteomes. Previously, 2D gel spots were excised manually or using a spot-picking robot [51,52,53,54,55,56,57]. More recent efforts have involved manual cutting of the gel into large grid sections [38,39]. However, neither approach is wholly practical or necessarily unbiased, and both are labour intensive, time consuming, and prone to human error or other variability. A significant analytical bias is exerted when only visually detectable spots are excised, which itself is dependent on the stain and imaging instrumentation used. As such, proteoforms below the limit of detection of the imaging are not analysed, despite increasingly sensitive staining protocols coupled with third separations (i.e., 3DE) and deep imaging to enable detection of lower-abundance species [19]. To address this and thus more broadly characterise proteoforms, including those below the limit of staining detection, gel sectioning, similar to slicing 1D gels into sections for deeper proteogenomic analysis, has been adopted by at least one research group [58,59]. Here, to streamline and semi-automate the process and thereby increase throughput while also reducing any potential cutting biases, a 3D-printed grid-cutting lattice was designed to the exact dimensions of the walls of a deep welled 96-well plate. This cutting device enabled the simultaneous and uniform sectioning of an entire mini gel (i.e., 7 × 7 cm) into 63 squares, effectively enabling extraction of every resolved proteoform. Aligning the grid to well-resolved high-abundance spots as reference points, similar to densitometry software for 2DE analysis [60,61], ensured reproducible alignment; between gel replicates, proteoforms appear in the same grid/well coordinate, removing variability and any likelihood of the same proteoform appearing in different wells. For an entire mini gel, this yielded 63 parallel samples for proteoform digestion and LC-MS/MS, ensuring reproducibility and a simplified, consistent downstream analysis process directly adapted to a 96-well format (Figure 1).

iTDP is well established as a robust tool for comprehensively detecting and identifying proteoforms from complex proteome extracts [10,11,13,20,21,25,29,31,32,33,38,57,62,63,64,65]. Indeed, the robustness of the 2DE analytical approach, both qualitatively and quantitively, between labs, and across two decades is highlighted by this assessment of the mouse liver proteome [29], The aim of the current proof-of-principle analysis was to assess the capacity of iTDP for still broader and more thorough characterisation of proteoforms across the breadth of a complex native proteome. Genuine analytical depth arises through simplification of the analysis of individual samples. Here, this was achieved through the coupling of high-resolution front-end 2DE, separating proteoforms according to pI and MW, with thorough LC/MS/MS analyses to identify ORF products, characterise specific PTMs, and thus resolve and identify proteoforms. Despite the obvious quality and depth of data provided by well-established iTDP analyses, proteogenomic assessments have been far more widely utilised over the past ~25 years, largely due to the relative speed of data generation; that is, quantity over quality in terms of analytical depth, bypassing any focus on proteoforms as the critical constituents of proteomes. Of course, any improvements in proteogenomic analyses also benefit iTDP, as it is the backend of the integrated analytical process [1,3]. Nonetheless, as 2DE and manual spot excision are often deemed labour intensive, here a new grid-cutting tool aligned with a 96-well plate, enabled rapid, reproducible processing of an entire 2D gel, reducing both manual labour and bias, and thereby also enabling detection and identification of proteoforms that would normally be missed by routine imaging. As grid cutting includes apparently ‘empty’ gel locations, a significant additional number of proteoforms were found (Figure 4, Figure 5 and Figure 6; Supplementary Data File S2). For example, grid coordinate C8 (Figure 1B) was a seemingly empty location with no detectable protein staining; however, numerous proteoforms were detected in this location, including Glutamate dehydrogenase-1, Aspartate aminotransferase and Malate dehydrogenase. Therefore, not only does the current approach capitalise on the more thorough sequence coverage afforded after proteoform resolution by 2DE [10,11,19,21,22,25,26,27,28,29,30,31,32,33,34,35,36,37,64] but it (i) expanded the range of proteoforms detected relative to more routine spot-cutting approaches and (ii) enabled detection of proteoforms that proteogenomic approaches are inherently blind to.

### 4.1. Enhanced Coverage and Reproducibility

Comparing iTDP and proteogenomic/shotgun workflows revealed that iTDP consistently identified a greater number of ORF products (Figure 2). Importantly, this difference held when identification criteria were extended to require ≥2 peptides, confirming that iTDP provided enhanced proteome coverage, more confident species identifications, and improved detection of lower abundance or otherwise difficult-to-detect proteoforms (see Section 4.5).

This result is perhaps not surprising. Theoretically, if 100 μg of a total protein extract is resolved on a 2D gel, then sectioning into 63 identical grids should result in an average of ~1.5 μg of protein per grid for analysis (assuming the protein load is evenly distributed). This is significantly higher than the 0.5 μg of peptides typically analysed using proteogenomics. However, peptide extraction efficiency from in-gel trypsin digests is ~70–90%, not including peptides lost to downstream handling processes [52,66,67]. If we conservatively consider 50% peptide recovery, ~0.75 μg of gel-extracted peptides is subjected to LC-MS/MS; this would increase detection per gel grid relative to a straight shotgun LC-MS/MS analysis as that 0.75 μg contains only a small subset of the total proteome that was resolved to the grid coordinate. Thus, like excised spots, this lower distribution of species at a greater concentration is ideal for MS analyses, yielding higher quality data (i.e., enhanced sequence and PTM coverage). Nonetheless, while it is clear that the total protein load is not uniformly distributed throughout the gel, the data also clearly indicate that LC-MS/MS is sufficient to identify even those proteoforms in regions of the gel having no visually detectable species despite use of a high-sensitivity staining protocol [32,42,68]. This further indicates the comprehensiveness and thus power of the hybrid iTDP approach that combines the two highest resolution approaches to proteome analysis at the critical level of proteoforms.

With an ~200× increase in total loaded protein using 2DE (i.e., 100 ug of total protein on a 2D gel vs. 0.5 ug of total peptides per shotgun injection), there is an expectation of a substantial increase in the detection of lower-abundance proteoforms using the iTDP analysis. However, despite a significant increase in ORF product and peptide identifications, the number of detected ORF products in this initial iTDP grid analysis did not outpace the proteogenomic approach to that extent. Since it is known that spot picking after deep imaging yields high-quality IDs of low-abundance species [19], it is likely that, because grid-sections are larger, some proteoforms are of too low abundance for the LC-MS/MS analysis. This is also a possible reason why some ORF products (i.e., 15%; Figure 2D) may be detected in a shotgun workflow but not in the grid-based iTDP analysis, as peptides common to all proteoforms (that separate to different 2D gel grids) elute together and overcome the limit of detection, being assessed only as single molecular entities in a proteogenomic analysis. While perhaps a potential limitation in this initial proof-of-principle assessment of a complete proteome using the grid-based approach, even without further optimisation, the data nonetheless clearly indicate that iTDP yields an overall deeper, more comprehensive profile of complex native proteomes at the general level of ORF products and, more importantly, at the critical level of proteoform diversity. The issue is thus one of analytical depth and quality, rather than simply inferring more canonical ORF product identifications.

The superiority of the iTDP method became more apparent when comparing the diversity of peptides identified by each approach. This is critical for proteoform-based analysis, as higher sequence coverage is essential for confidently assigning modification profiles and distinguishing between proteoforms using unique combinations of modified and unmodified peptides. This was particularly evident when interpreting protein groups, effectively highlighted by the protein Histone H3.3 and a peptide for which the iTDP data were sufficient for confident assignment as the ORF product present in the sample, rather than variant species (Supplementary Table S1). This further emphasises how improved sequence coverage directly increases confidence in identifying actual proteoforms (i.e., rather than simply inferring the presence of intact canonical proteins) [69,70].

On a proteome-wide scale, prefractionation/proteoform concentration by 2DE results in lower diversity of peptides simultaneously eluting in LC-MS/MS, thereby significantly increasing the signal of individual species, yielding increased canonical sequence coverage and enabling the identification of proteoforms. Average sequence coverage was ~37% higher by iTDP, and ~75% higher when comparing only commonly identified proteins (Figure 3); thus, front-end proteome fractionation by 2DE enhances proteoform-level proteome coverage by improving the resolution and confident identification of proteoforms, including size variants, rather than simply assuming the presence of an intact canonical protein. This increased proteome coverage was achieved through an increase in the dimensions of separation, first using 2DE based on defining physio-chemical properties of proteoforms, followed by separation of constituent peptides; in contrast, a standard proteogenomic experiment only uses peptide separation afforded by the chosen chromatography.

### 4.2. Detection of Proteoforms

2DE is uniquely suited for high-resolution, truly comprehensive, in-depth proteoform-level analysis, across the breadth of a native proteome, due to its inherent capacity to separate proteoforms according to the orthogonal physio-chemical properties of net charge and MW. As these properties are unique to different proteoforms, individual proteoforms originating from the same ORF product resolve to distinct gel locations (i.e., intersecting coordinates of pI and MW) and can be analysed in separate LC-MS/MS injections. This spatial separation thus provides direct linkage between a detected modification and its associated proteoform. Furthermore, significant shifts in canonical MW and/or pI of an identified ORF product confidently confirm the presence of a unique proteoform without knowledge of the modification(s) yielding the specific proteoform. This level of resolution and confidence in ID is inherently unattainable using proteogenomic workflows, as peptides are disconnected from their parent proteoforms. As a consequence, iTDP detected a substantially greater diversity of PTMs, identifying 78 distinct modification classes, compared to only 23 by proteogenomics (Supplementary Table S2). Beyond class diversity, more modified peptides were also detected overall without the need for separate enrichment strategies and analyses. For phosphorylation (STY), iTDP identified 94 modified peptides vs. only 16 by proteogenomics. Similarly, for acetylation, iTDP identified 166 modified peptides vs. only 23 by proteogenomics (Supplementary Figure S3). Numerous other biologically important modifications, including methylation, sulfation, and ubiquitination, were consistently and reproducibly detected across the iTDP replicates but were absent or poorly detected using proteogenomics (Figure 5).

Part of the power of the iTDP approach then is that front-end 2DE resolves modified ORF products to discrete gel coordinates, providing spatial evidence to complement and confirm proteoform IDs by subsequent LC-MS/MS (Figure 4). For Nucleobindin-1, a phosphorylation at residue S368 was detected, which has not been previously reported in murine samples in the UniProt database, though a homologous site exists at residue S369 due to a frame shift mutation in the human orthologue. Similarly, an acetylated proteoform of GDH-1 resolved to a single high-abundance location (grid B6 across all three replicate gels; Figure 5), with additional resolved proteoforms identified across the pI and MW ranges. Shifts in the pI of an ORF product are typically due to the presence of modifications; however, the other proteoforms of GDH-1 detected in other grids yielded no modified peptides. When a proteoform resolves to a grid coordinate different from its theoretical MW or pI, and no PTM was identified, it is likely that the modifications either evaded MS detection for numerous documented reasons, including low abundance or poor ionisation efficiency (see Section 4.5), or the lability of the modifications resulted in their loss during post-2DE processing and/or LC-MS/MS. Nonetheless, a shift in pI and/or MW is consistent with identification of a specific proteoform, and such information alone, gleaned solely from the 2D gel, is notable when trying to identify critical molecular entities such as biomarkers or drug targets [3,5,10,44,71,72,73,74].

Nucelobindin-1 (NUCB1) (Figure 4) also showed evidence of a size variant, possibly due to proteolytic cleavage, as a proteoform was consistently detected at grid coordinate F4 (~18 kDa), well below the intact mass of the canonical protein. Based on the data, the sequence of NUCB1 at grid F4 ends at residue R96. This was an important finding since there is no corresponding cleavage site at that location that has been reported in current databases. This may thus represent a novel NUCB1 isoform or a cleavage product; further study is required.

Albumin, much like GDH-1, displayed a broad distribution across gel coordinates (Figure 6). Applying a minimum sequence coverage threshold of 5% revealed an array of low coverage proteoforms primarily at low MW regions, likely cleavage products, distributed across the gel. Increasing the coverage threshold to 40% revealed a dominant set of high MW proteoforms between 75 and 250 KDa (Figure 6). This resolution and detection of proteoforms is consistent with our previous work on purified BSA that highlighted proteoform heterogeneity in commercially purified samples [44].

### 4.3. Protein Complexes

Despite the expectation that the chaotropes, surfactants and reducing agents used in protein extraction protocols are sufficient to yield linearized single molecules of every ORF product in solution and dissociate them from all other molecules (i.e., potential binding partners or interactors), it is well established that certain protein complexes and interactions (e.g., oligomers or aggregates) require specific conditions to achieve full dissociation and monomerization [75,76,77,78]. Several complexes have been shown to resist disruption or form additional non-native complexes in the presence of strong denaturants such as urea and SDS, instead requiring heat-induced unfolding for complete monomerization or other treatments such as high concentrations of denaturant [79,80,81]. However, the resolution of native complexes should also be recognised as an important part of assessing functional components of native proteomes by iTDP. The complications lie in discerning the native complexes from the artefactual.

The phenomenon of stable complexes was thus also evident in this study. Despite the 2DE workflow employing 7 M urea, 2 M thiourea, and 1% C7BzO during protein extraction, followed by reduction (5 mM TCEP) and alkylation (10 mM acrylamide) and 6 M urea with 2% *w*/*v* SDS during IEF strip equilibration to maintain solubility and ensure individual proteoforms were denatured and remained linearized, some species were routinely resolved and detected in MW regions far exceeding the theoretical monomer mass of the corresponding ORF product. A notable example was GDH-1 with a canonical MW of 61.3 kDa, which was nonetheless detected across multiple gel coordinates (Figure 5) with proteoforms spanning from 15 to 250 kDa. Cross-linking MS data has shown GDH-1 to form a stable homohexamer, and its dissociation under denaturing conditions to proceed in a stepwise manner, via intermediate trimeric complexes [82]. When examining proteins known to interact with GDH-1, including Malate dehydrogenase and Aspartate aminotransferase [83], several were detected with overlapping spatial distribution across the gel (Figure 5), suggesting possible co-migration due to persistent interaction or complex retention [49,50,84]. Furthermore, intrinsically disordered regions in proteins, which exist in at least one third of eukaryotic proteins and may be influenced by PTMs, can result in migration to higher MW regions during gel electrophoresis due to retained charge and/or polarity [85,86].

Full denaturation of different mammalian protein complexes has been shown to require boiling in strong extraction buffer, such as one containing SDS, to overcome the resistance to denaturation [87,88]. However, IEF requires non-charged denaturants and incorporating a boiling step into a standard 2DE workflow is problematic as elevated temperatures accelerate the decomposition of urea into cyanate, which induces artifactual carbamylation of proteins and has the potential to negatively impact labile PTMs [89,90,91]. While some artifactual modifications are typically included in MS searches, the same PTM can also be biologically relevant; therefore, it is obviously preferable to prevent or minimise the induction of such artefacts, especially when the modification is unlikely to occur in 100% of potential locations across all proteoforms in a sample. An increase in artifactual modifications creates artifactual proteoforms, which reduce the abundance of the true proteoform(s) and decrease effective detection. An alternative approach of extracting proteoforms in a high heat-compatible buffer such as SDS or sodium deoxycholate followed by precipitation with an organic solvent such as acetone and resuspending the precipitated protein pellet in a 2DE-compatible buffer has been proposed [92]. However, together with the high heat, precipitation carries its own limitations. Organic solvent precipitation disrupts the hydration layer that helps keep proteins in solution, promoting the formation of strong intra- and intermolecular forces that drive protein aggregation and refolding, potentially reintroducing the same or additional types of problematic interactions [93,94,95]. Furthermore, there is also a potential to selectively and/or non-specifically lose species to the organic solvent and to induce additional artifactual modifications; however, this has not been extensively assessed or characterised in the literature [96].

Additionally, GDH-1 was detected in the entire MW region, including MW grids considerably below its monomer mass (61.3 kDa), with the lowest detection at ~15 kDa (Figure 5). Fragmentation of GDH into ~44 kDa and ~15 kDa fragments has been previously described for human GDH and attributed to shearing at a flexible “hinge loop” bridging the two structural domains [97]. This loop is integral to the catalytic mechanism of the active protein. It has been documented that sonication-induced acoustic cavitation can generate shear forces that place mechanical strain directly on proteins, with a plausible point of stress for GDH-1 being this hinge loop. During sample preparation, the sonolysis of water can also produce free radicals capable of cleaving the already-stressed peptide bonds at this site [98,99,100,101,102,103,104], a phenomenon not tested in this work. The result is domain-level fragmentation that occurs independently of, and potentially despite, intact protein–protein interactions elsewhere in the complex. While analyses specifically targeting protein complexes or interaction networks should employ non-denaturing strategies such as native 2D-PAGE or diagonal 2D-PAGE [105,106,107], it is important to acknowledge that under standard denaturing conditions, some interactions are sufficiently stable to persist through sample preparation and may manifest as apparently anomalous gel migrations that warrant careful interpretation; these may in fact be informative of native molecular interactions and mechanisms.

Finally, a diffuse low-intensity signal, referred to as “the cloud”, has been observed in Mycoplasma as a ‘background’ across Western blotted 2D gels probed with convalescent sera. This has been hypothesised to represent a population of low-abundance cleavage fragments rather than artefacts [55]. The present data also suggest that this “cloud” may encompass low-abundance proteoforms and variations in protein complex constituents. Although beyond the scope of this study, definitive validation of cleavage-derived proteoforms, including those observed here for GDH-1 and Nucleobindin-1, could be achieved through N- and C-terminal labelling strategies to confirm proteoform boundaries prior to tryptic digestion [108].

### 4.4. MS Detection Challenges

Following precursor ion selection during the MS1 scan of a DDA cycle, peptides are isolated and subjected to fragmentation prior to determining amino acid sequences in the MS2 scan. This requires enough energy to be imparted to the precursor to cause the peptide bonds to break, with the most common forms being Collision-Induced Dissociation (CID) or High-Energy Collisional Dissociation (HCD). While these fragmentation methods have demonstrated their utility in peptide backbone bond fragmentation to determine sequences, they have also been proven to be unsuitable for detecting a range of PTMs. This is largely due to the bond between amino acids and their respective modifications often being weaker than peptide bonds. This results in the amino acid-to-modification bond being preferentially cleaved during peptide fragmentation, resulting in loss of the modification and thus absence of a localised modified fragment ion in the MS2 scan. In such cases, the MS1 scan can indicate a mass shift induced by a modification, but direct MS2 evidence and ion intensity are lost. This should not be an indication to disregard the modification, but it does highlight that spectra must be manually evaluated and interpreted with specific attention regarding diagnostic ions and neutral losses [109,110,111]. Again, the benefit of having pI and MW information from the 2D gel can be used to guide further analysis, particularly since PTM-specific stains may also be utilised (e.g., phospho-specific staining may assist in determining if labile phosphorylations have been lost) [22,44,112,113]. Further complementing this, computational tools such as PEAKS Studio and Fragpipe employ probability algorithms to determine likely modification locations in the absence of direct fragment ion evidence [114,115,116].

To overcome the limitations of CID with regard to PTM identification, electron-based fragmentation methods, such as Electron Transfer Dissociation and Electron Capture Dissociation, offer alternatives that may preserve labile PTMs, while still fragmenting the peptide backbone. However, these approaches have exhibited lower fragmentation efficiency than traditional CID-based approaches, and therefore should be used as a supplement to CID-based fragmentation or employed during a hybrid strategy such as Electron Transfer/High-Energy Collision Dissociation or Electron-Activated Collision-Induced Dissociation [117,118,119,120].

Compounding these issues, numerous PTMs have been shown to directly influence electrospray ionisation efficiency [121,122,123,124]. Modifications such as phosphorylation and acetylation reduce the number of basic sites available for protonation, thereby suppressing ionisation efficiency of the modified peptide. Beyond their potential sheer complexity, glycosylation also presents a different yet equally problematic issue, with the hydrophilic nature of glycopeptides causing them to partition towards the interior of ESI droplets, thereby reducing their likelihood of protonation and ejection as gas-phase ions relative to the more hydrophobic peptides at the droplet surface. Conversely, some modifications such as dimethylation have been shown to enhance ionisation efficiency due to the modification allowing the peptide to maintain a net positive charge [123]. Overall, this highlights that modifications can impart complex and specific effects on downstream MS analysis.

Taken together, these challenges have meaningful implications for proteoform-level analysis, as PTMs are often what define unique proteoforms of the same or similar ORF products. With individual gel spots routinely yielding up to hundreds of distinct proteoforms and the dynamic range of proteoform abundance in a complex sample spanning up to 12 orders of magnitude, it is likely that a substantial fraction of the proteome remains below the detection threshold, even with routine 2DE fractionation [21]. While higher sample loads and even higher-resolution 2DE approaches can help address this to some extent [11,125,126], it should be noted that critical limitations to the grid-iTDP methodology used to generate the data here arise from MS instrumentation, and that the inherent limitations encountered are not related to the 2DE portion of iTDP. Indeed, these instrumentation limitations are common to all proteomic analyses regardless of the prior separation methodology employed. Lower-abundance proteoforms, particularly those carrying modifications that suppress ionisation or are not overly compatible with CID, may fall outside the capabilities of current instrumentation. However, emerging high-sensitivity platforms may offer the resolution and sensitivity required to access these lower-abundance species and further close the gap in proteoform-level coverage [127,128,129].

### 4.5. Limitations and Future Directions

The current proof-of-principle study utilised a mini-gel format in order to balance resolution with analytical time at the potential expense of complete resolution of a single proteoform from all others, something not routinely achieved in proteomics. To address this issue, larger first and second dimension gels, with larger total protein loads, can be used to increase resolution of individual proteoforms [11,125,126]. However, this would result in a proportionally increased analytical time. Alternatively, or perhaps in addition, a cutting grid yielding smaller gel sections (e.g., to overlay on a 384-well plate) would also increase resolution of individual proteoforms as they would occur in different LC-MS/MS analyses. However, this would again result in a proportional increase in instrument time. Recently reported approaches to enhancing LC-MS/MS throughput without (markedly) detracting from the quality of peptide analyses could also help address increases in grid numbers [127,128]. In short, proteoform-level proteome characterisation requires lengthy time-frames to comprehensively generate the necessary data.

Additionally, as advancements in DIA analysis software progress, this approach becomes increasingly more viable for PTM analysis. Currently, DDA remains the preferred/primary acquisition method due to its improved site localisation of modifications [130]. Narrow window (2 *m*/*z*) DIA (nDIA) has the advantage of being unbiased in peptide selection while producing pseudo DDA MS2 spectra as it is highly unlikely that different yet isobaric peptides will have the same chromatographic retention time. This results in more peptides in the dataset as no selection is occurring with an increasing number of PTM-containing peptides waiting to be characterised [131,132]. With the reduced sample complexity produced by iTDP, nDIA should characterise more peptides and their PTMs, increasing the confidence of proteoform identifications. Currently, there exists a plethora of software packages designed to assist with the interrogation and interpretation of proteogenomic data. In contrast, there are few programmes, such as Proteoform X (Bioinformatics Solutions), designed to interrogate and interpret iTDP-based datasets. This results in sole reliance on manual evaluation and interrogation of data and thus potentially associated biases. Further work is thus needed to improve the interrogation and analysis of proteoform-based datasets, with critical attention to iTDP analyses, including the ability to compare intra- and inter-gel proteoform identifications.

## 5. Conclusions

This proof-of-principle study revealed the breadth of proteoform complexity within a mammalian tissue proteome. iTDP, leveraging the capacity of 2DE to highly resolve individual proteoforms via their unique pI and MW, enabled definitive identifications of ~29,000 unique proteoforms, in direct contrast to the generalised inference of ORF products that has become routine with proteogenomics. The new grid-cutting approach enabled a rapid, unbiased, fully reproducible sectioning of replicate 2D gels, interfaced with established parallel in-gel digestion and high-resolution LC-MS/MS, and yielded wider detection and characterisation of proteoforms, including those that would otherwise have been missed in regions of the gel visually devoid of stained species. Overall, relative to standard proteogenomic analysis, iTDP provided an unbiased increase in the detection of modified peptides, ORF products, and critically, through preserved pI and MW information, definitive proteoform-level analyses. Thus, this grid-cutting iTDP approach is presented as a logical step forward to broadly characterise proteomes at the critical level of proteoforms.

## Data Availability

The mass spectrometry data have been deposited to the ProteomeXchange Consortium via the PRIDE partner repository with the dataset identifier PXD081275.

## Supplementary Materials

The following supporting information can be downloaded at: https://www.mdpi.com/article/10.3390/proteomes14040053/s1, **Supplementary Figure S1**: Gel images of resolved mouse liver via 2D-PAGE stained for total proteoform detection with Coomassie Brilliant Blue used for iTDP analysis; **Supplementary Figure S2**: A visual representation of the gel slicing workflow; **Supplementary Table S1**: Histone H3.3 Isoforms from one Proteogenomics sample compared to one iTDP where ND signifies Not Detected; **Supplementary Figure S3**: Number of selected post translational modified (PTM) peptides identified by Peaks Studio in the proteogenomics and iTDP data; **Supplementary Table S2**: Summary table of number of post translational modified peptides identified by Peaks Studio in the proteogenomics and iTDP data where ND signifies Not Detected. SupplementaryDataFile_S1 and SupplementaryDataFile_S2.
