# Supplementary material for: Integrated Top-Down Proteomics for Comprehensive Proteome Analysis at the Proteoform Level
Source: Proteomes. 2026 Oct 9;14(4):53. doi: 10.3390/proteomes14040053 (PMC13692341; doi:10.3390/proteomes14040053)
Supplement: Supplementary file 1 [file proteomes-14-00053-s001.zip › SupplementaryData.pdf]

Supplementary Data

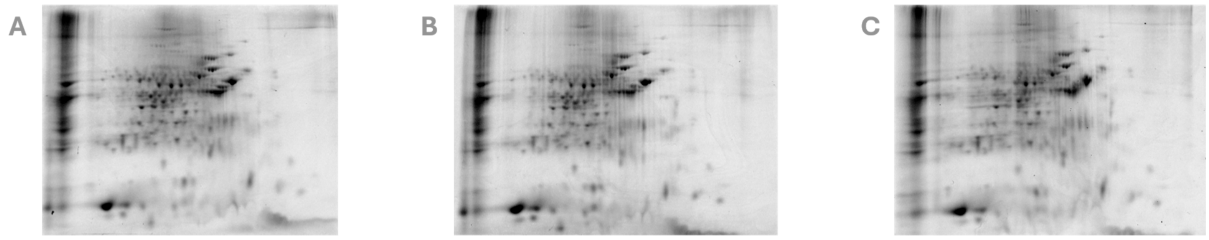

Supplementary Figure S1. Gel images of resolved mouse liver via 2D-PAGE stained for total proteoform detection with Coomassie Brilliant Blue used for iTDP analysis.

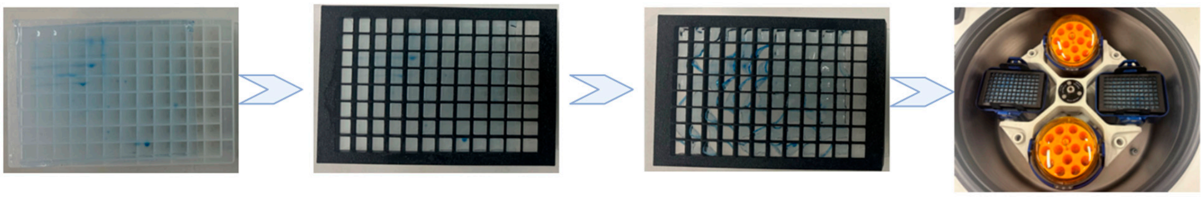

Supplementary Figure S2. A visual representation of the gel slicing workflow. The 2D gel gently placed on top of the deep 96-welled plate covering 63 grids/wells in a 9 x 7 configuration (image one). The gel slicer is then aligned and placed on top, gently resting and holding the gel in place (image two). The gel slicer is then gently pressed onto the gel, slicing the gel into 63 squares by sandwiching between the walls of the 96 well plate (image three). The entire deep welled plate combination is then transferred into a centrifuge and spun the pull and gel squares into the bottom of the gel (image four).

Supplementary Table S1. Histone H3.3 Isoforms from one Proteogenomics sample compared to one iTDP where ND signifies Not Detected

	**Proteogenomics**			**iTDP**		
Accession Number	Confidence	Sequence Coverage (%)	# Unique Peptides / # Peptides	Confidence	Sequence Coverage (%)	# Unique Peptides / # Peptides
P84244	True	31.62	2/2	True	68.38	9/9
E0CZ27	True	36.13	2/2	True	78.15	9/9
F8WI35	False	8.15	1/1	False	45.19	8/8

P02301	True	31.62	2/2	False	61.76	8/8
P84228	False	8.09	1/1	False	37.5	7/7
P68433	False	8.09	1/1	False	37.5	7/7
A0A8I4SYN6	False	8.09	1/1	False	24.26	5/5
E0CYN1	ND	ND	ND	False	71.15	5/5

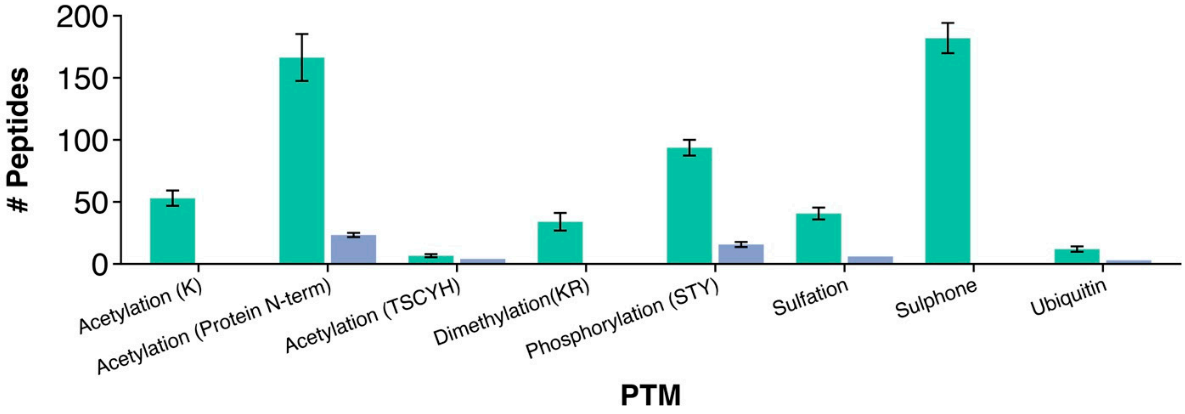

Supplementary Figure S3. Number of selected post translational modified (PTM) peptides identified by Peaks Studio in the proteogenomics and iTDP data. Selection was chosen based on the PTM being of increased interest due to their known ability to cause changes in a proteoform's function, and are not artifactual modifications.

Supplementary Table S2. Summary table of number of post translational modified peptides identified by Peaks Studio in the proteogenomics and iTDP data where ND signifies Not Detected.

Post Translation Modification (PTM)	# Peptides (Average ± SD)	
	Proteogenomics	iTDP
2-amino-3-oxo-butanoic_acid	ND	111 ± 11
4-hydroxynonenal (HNE)	2 ± 0	3 ± 1
4-Oxononenal (ONE)	ND	5 ± 2
5-dimethylaminonaphthalene-1-sulfonyl	ND	2 ± 0
Acetaldehyde +26	ND	8 ± 0
Acetylation (K)	ND	53 ± 6
Acetylation (N-term)	ND	56 ± 8
Acetylation (Protein N-term)	23 ± 2	166 ± 19

Acetylation (TSCYH)	4	7 ± 1
Amidation	ND	40 ± 2
Amidination of lysines or N-terminal amines with methyl acetimidate	6	ND
Aminoethylbenzenesulfonylation	9	55 ± 5
Ammonia-loss (N)	7 ± 3	71 ± 10
Beta-methylthiolation	ND	14 ± 0
Biotin polyethyleneoxide amine	ND	2 ± 0
Biotinylation	ND	17 ± 5
Carbamidomethylated Cys that undergoes beta-elimination and Michael addition of ethylamine	ND	2 ± 0
Carbamidomethylation (DHKE, X@N-term)	3	61 ± 11
Carbamylation	13 ± 2	60 ± 14
Carboxyl modification with ethanolamine	3	ND
Carboxylation (DKW)	ND	13 ± 0
Carboxylation (E)	ND	20 ± 0
Carboxymethyl (KW, X@N-term)	ND	6 ± 1
Cyano	ND	159 ± 18
Cysteine oxidation to cysteic acid	ND	19 ± 4
Deamidation (NQ)	461 ± 28	2694 ± 89
Deamidation (R)	ND	13 ± 0
Deamidation followed by a methylation	ND	13 ± 2
Dehydration	32 ± 13	278 ± 58
Dehydroalanine (C)	12 ± 2	119 ± 71
Deoxy	ND	20 ± 4
Dethiomethyl	ND	4 ± 3
Dihydroxy	ND	129 ± 13
Dimethylation(KR)	ND	34 ± 7
Double Carbamidomethylation (DHKE, X@N-term)	ND	12 ± 1
Ethyl amino	1	5 ± 0
Ethylation	ND	18 ± 0
Formylation	ND	62 ± 9
Formylation (Protein N-term)	ND	10 ± 1
Glycidamide adduct	ND	77 ± 19
Guanidination	ND	20 ± 7
HexNAcylation (N)	ND	14 ± 0
Hexose (NSY)	ND	34 ± 10

Levuglandinyl - lysine hydroxylactam adduct	ND	1 ± 0
Lysine oxidation to aminoadipic semialdehyde	ND	19 ± 0
Methylation(KR)	12	106 ± 2
Methylation(others)	6	251 ± 22
Methylmalonylation on Serine	ND	2 ± 0
Methylpyrroline	ND	1 ± 0
Myristoylation	ND	4 ± 2
N-Succinimidyl-2-morpholine acetate	9 ± 2	5 ± 0
Naphthalene-2,3-dicarboxaldehyde	ND	6 ± 0
O-Diisopropylphosphorylation	ND	7 ± 3
O-Ethylphosphorylation	ND	2 ± 0
Oxidation (HW)	ND	153 ± 11
Oxidation (M)	80 ± 5	4927 ± 274
Oxidation or Hydroxylation	ND	21 ± 3
Oxidation or Hydroxylation (C)	ND	12 ± 4
Phenethyl isothiocyanate	ND	4 ± 3
Phosphorylation (STY)	16 ± 2	94 ± 6
Phosphorylation to amine thiol	ND	31 ± 0
Proline oxidation to pyroglutamic acid	ND	9 ± 4
Proline oxidation to pyrrolidinone	ND	11 ± 0
Propionaldehyde +40	26 ± 0	4 ± 0
Propionamide	1076 ± 40	2488 ± 91
Propionamide (K, X@N-term)	ND	46 ± 10
Pyro-glu from E	ND	97 ± 5
Pyro-glu from Q	36	183 ± 11
Pyrrolidone from Proline	ND	2 ± 0
Quinone	ND	6 ± 2
Replacement of 2 protons by nickel	ND	28 ± 2
S-pyridylethylation	ND	66 ± 0
Sodium adduct	ND	33 ± 0
Sulfation	6	41 ± 5
SulfurDioxide	ND	15 ± 1
Sulphone	ND	182 ± 12
Tryptophan oxidation to kynurenin	ND	89 ± 25
Tryptophan oxidation to oxolactone	ND	3 ± 1
Tyrosine oxidation to 2-aminotyrosine	ND	8 ± 0
Ubiquitin	3	12 ± 2
